# Integrating Sustainability into Biologically Inspired Design: A Systematic Evaluation Model

**DOI:** 10.3390/biomimetics10020111

**Published:** 2025-02-12

**Authors:** Ze Bian, Yufei Zhang, Huan Lin, Yuan Zhu, Jie Zhang

**Affiliations:** 1 School of Art and Archeology, Hangzhou City University, Hangzhou 310025, China; bianz@hzcu.edu.cn (Z.B.); zhuyuan@hzcu.edu.cn (Y.Z.); 2 College of Mechanical Engineering, Quzhou University, Quzhou 324000, China; linhuan_design@zju.edu.cn; 3 Faculty of Applied Sciences, Macao Polytechnic University, Macau 999078, China

**Keywords:** biologically inspired product design (BIPD), sustainability, analytic hierarchy process (AHP), design evaluation

## Abstract

Biologically inspired product design (BIPD) inherently encompasses the concept of sustainability. It acquires inspiration from natural organisms, and the references in aspects such as form, structure, and function typically contribute to efficient resource utilization and environmentally friendly coexistence. However, past studies have mainly evaluated from the perspective of designers and researchers, which is relatively subjective. It is difficult to meet the real needs of industry and market. At the same time, the method of establishing indicators is not scientific enough, and the importance of indicators is not ranked. This research integrates the concept of sustainable design into the BIPD evaluation system, comprehensively considering the evaluation indices of different stakeholders such as sustainable designers, industrial designers, and users and decision-makers of design companies. By employing the analytic hierarchy process, a complete and systematic evaluation index model is constructed. This model can comprehensively and accurately screen and evaluate design proposals during the conceptual design stage of BIPD. Through this approach, it effectively averts resource waste caused by incorrect decisions in the production process, optimizes resource allocation, meets user requirements and vigorously promotes the sustainable development of BIPD throughout its entire life cycle.

## 1. Introduction

Biologically inspired design (BID), also known as bionics design or biomimicry, is a design-by-analogy method that draws inspiration from nature [1,2]. It utilizes the shape, material, structure, and principles of biological organisms in diverse design fields such as engineering design [3], mechanical design [4], architectural design [5], industrial design [6], and other design fields [7,8]. Biological systems have evolved over millions of years to optimize resource utilization, energy efficiency, and adaptability, which are essential aspects of sustainable design [9,10,11]. Bio-inspired design, in which the target domain is a product and the source domain is biology, is called biologically inspired product design (BIPD). In BIPD, designers create products based on inspiration from the appearance, structure, and behaviors of living organisms [12,13].

Sustainability typically encompasses economic development, social inclusion, and environmental sustainability. The design discipline can play a significant role in achieving these goals by rethinking industrial products, processes, and broader business operational modalities [14,15]. Sustainable design refers to the process of designing to address issues related to sustainability [16]. Existing sustainable design places greater emphasis on numerous considerations from a product’s design to the end of its life cycle [17], especially the sustainable factors of products in the commercial realm [16]. The function of sustainable design theory is to transform products, human behaviors, commercial services, cities, and ultimately the entire socio-economic system [18].

Sustainability evaluation, also known as sustainability assessment, is often described as the process of assessing the impact of an initiative on sustainability [19]. The aim of sustainability assessment is to offer decision-makers an integrated and systematic appraisal from both short-term and long-term standpoints, aiding them in determining which actions should or should not be adopted to strive for social sustainability [20]. Numerous scholars have delved into the methods and instruments for sustainable assessment [21], and typically a multitude of aspects must be taken into account, such as economy and market, innovation and technological progress, industry ecology, and product life cycle [22]. Yonat et al. [23] considered design errors as social and psychological phenomena and proposed a design management theoretical framework and practical methods for controlling design risks in complex information systems.

With the rapid development of generative artificial intelligence, large models and generative AI tools like Midjourney, DALL-E, and Stable Diffusion have become popular [24,25,26]. A large number of BIPD solutions can now be generated in a short time. In this context, the importance of evaluation becomes even more pronounced.

In BIPD, a comprehensive and sustainable assessment is essential before starting production. This assessment aims to select the most appropriate design solution to meet multiple key aspects, with the objective of reducing waste and decreasing energy consumption [27,28]. First, it must align with user preferences. If consumers do not prefer the sustainable products over traditional alternatives, it will be very difficult to achieve actual environmental improvements [29]. Additionally, the product must conform to long-term aesthetic expectations. Some products might gain consumers’ short-lived favor but ultimately fail to endure the test of time. Either due to low usage frequency or being phased out by the market in the future, such a situation also represents a form of unsustainability [30,31]. Moreover, it has to fulfill production requirements. This involves considerations such as the availability and cost of materials, the complexity and feasibility of manufacturing processes, and the overall production efficiency [32,33].

Certainly, the above-mentioned considerations are insufficient to form a complete BIPD evaluation model. When adding sustainability to the numerous issues that the manufacturing industry needs to consider, industrial designers usually do not have sufficient capabilities to provide advice [34]. Therefore, it is necessary to invite more stakeholders to collect BIPD indicators so as to build a more scientific and comprehensive evaluation system [35].

This paper tries to establish a sustainable evaluation system for BIPD with indicators from different stakeholders including sustainable design experts. By comprehensively considering user needs, production requirements, biological design features, and so on, a scientific, systematic, and effective evaluation model is developed. Finally, the effectiveness of the model is verified through the evaluation calculation of a traditional case and an artificial intelligence-generated content (AIGC) case. This model not only serves to evaluate and screen design solutions but also acts as a guideline for designers in the initial stage of design. It encourages designers to make comprehensive and balanced considerations regarding BIPD and avoid the waste of resources due to wrong decisions.

## 2. Related Works

### 2.1. BIPD Evaluation Indicators

Previous research on biologically inspired design (BID) evaluation was often conducted from the perspective of design experts, who formulated bionic evaluation indicators through discussion. The metrics were usually related to the purpose of their research.

In biologically inspired engineering product design, quantity, variety, novelty, similarity, and quality were chosen as metrics to evaluate a BID product [36,37,38]. The purpose of constructing these evaluation indicators was to explore the impact of different examples and materials on BID output, aiming to find better ways to improve designers’ performance.

These metrics were derived from common design process studies in which designers’ performance and output were the objects of evaluation. The number of solutions, originality, practicality, quality, detail, and flexibility are common evaluation indices in design process studies [39,40,41]. These evaluation indicators include both subjective and objective evaluations. Shah et al. [42] and Nelson et al. [43] proposed that novelty can be calculated. The main idea is that the fewer times a certain solution appears, the higher its degree of innovation.

In addition to the expert perspective, subsequent studies began to focus on user perception preferences. Luo et al. [44] used the method of perceptual imagery to establish a model that can predict user preferences through the designer’s perceptual evaluations. Li et al. [45] constructed a data-driven intelligent service model for BID considering user perception needs based on Kansei engineering.

Zhu et al. [46] used the fuzzy rough number extended multi-criteria group decision-making method to evaluate and rank different biological inspirations and evaluate the risks of these inspirations before they are applied. But it mainly focused on BID in the engineering field and evaluated technical potential and effectiveness standards.

Table 1 lists the research contents, indicator sources, construction methods, and indicators of previous studies on BID evaluation and this study. Most of the previous evaluation indicators served other research purposes. and there is no research on establishing evaluation indicators specifically for BIPD. In addition, these indicators are mainly oriented towards design education or scientific research, rather than towards real industry needs. Moreover, the sources of these indicators are mainly limited to researchers themselves, designers, and ordinary users. Finally, these indicators do not have an importance ranking or hierarchical relationship.

Faced with the rapidly developing AIGC industry and the need for large-scale screening, there is still a lack of a more systematic, scientific, and rapid evaluation calculation method for screening out a sustainable design at the economic, social, and ecological levels. This study recruited different stakeholders in the industry to collect indicators and established an effective BID evaluation method through hierarchical analysis.

### 2.2. Analytic Hierarchy Process

This study uses the Analytic Hierarchy Process (AHP) method to construct a BIPD evaluation model. First, BIPD evaluation is a multi-objective decision-making problem that needs to consider multiple factors such as usability, cost, user preferences, bionic effects, and so on. There are also complex relationships between these factors. The AHP, developed by Saaty [48], is a multiple-criteria decision-making tool that has been used in many decision-making situations. AHP decomposes the decision-making problem into different hierarchical structures from the overall goal to sub-goals at each level, evaluation criteria, and specific investment plans. This structured method makes the decision-making process clearer and more organized, which is suitable for BIPD evaluation.

In addition, BIPD evaluation belongs to the design decision, many indicators are difficult to quantify, and it always relies on the experience of experts. Simultaneously, it is essential that the BIPD evaluation exhibits a certain level of scientific rigor. AHP is a combination of qualitative and quantitative methods. Experts obtain the weights of indicators through comparison matrices and avoid logical contradictions in the decision-making process through consistency tests.

The AHP method is commonly used to make design decisions in a variety of areas. Nukman [49] and Hambali et al. [50] explored the use of AHP for selecting the best design concept. Cambron and Evans [51] illustrated an AHP approach to the layout design problem of a commercial printing and binding facility, considering various difficulties. Lin et al. [28] applied AHP to construct an evaluation manual for tail-light shape design, including safety and aesthetics. Zhang et al. [52] used AHP to study the influencing factors of the intervention of graphic art in the rural cultural tourism space environment. Pan et al. [53] constructed an evaluation method for AIGC-generated cultural heritage design through AHP.

This study uses AHP to construct a BIPD evaluation system, organizes and categorizes different evaluation indicators, and decomposes them into three levels. It then obtains the weights between different indicators at the same level. The result of this study is a way to enable rapid and consistent product evaluations in multi-objective decision-making problems that typify BIPD.

When using AHP to construct a decision-making model, there are four steps, as follows [54,55,56]:

(1) Define the problem and collect criteria: The first step is to analyze the problem and identify its relevant indicators.
(2) Create a hierarchy: The second step is to analyze the relationships between indicators in the system and establish a hierarchical structure model for the system. This hierarchy consisted of different levels such as the overall goal level, sub-goal level, criteria level, and evaluation level.
(3) Importance pairwise comparison: For all indicators of the same level belonging to a certain criterion at the upper level, comparison matrices are constructed to compare the importance of every two indicators.
(4) Calculate the weights of each level: This step is to acquire the weights of each level. The judgment matrices obtained from step (3) can be used to calculate the relative weights of every indicator in each level.
(5) Calculate the overall weights of leaf indicators: After the relative weights of every indicator in each level have been calculated (step (4)), the final relative weights of all leaf indicators are calculated by multiplying the weights of the abovementioned several levels.

## 3. Methodology

**Figure 1:**
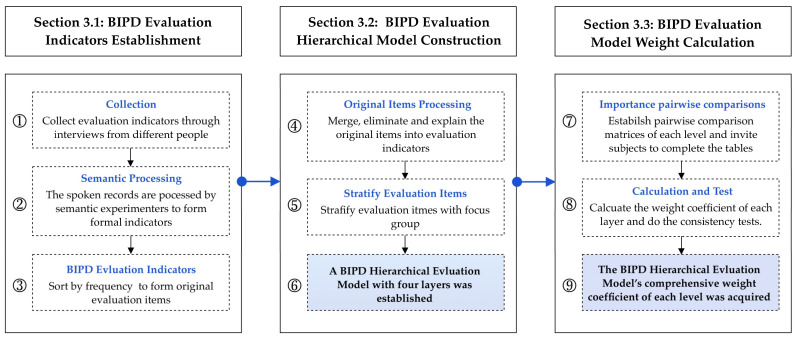
Our proposed pipeline of BIPD evaluation hierarchical model construction.

Three steps are required to construct a quantified hierarchical structure evaluation model for biologically inspired product design, as shown in Figure 1. The first step is the collection of BIPD evaluation indicators, including obtaining original evaluation items through interviews and merging synonymous indicators. The second step is the construction of a BIPD evaluation hierarchical model, which is established by focus groups of experts. The third step is the determination of the weights of the evaluation model. Importance pairwise comparisons of the criterion at each level are conducted by distributing questionnaires to experts. Then, the eigenvector method is used to calculate the weights and conduct a consistency test. The average weight of the subjects is taken as the final weight of the model.

### 3.1. BIPD Evaluation Indicators Establishment

#### 3.1.1. Experimental Design

(1) Subjects: In order to establish a comprehensive product bionics evaluation model oriented toward real design, production, and sales, different stakeholders should be involved in the experiment. A total of 16 subjects were invited to participate in the experiment. Among them, there were four senior product designers (two males and two females) with more than five years of product design experience, two sustainable design experts, one design company decision-maker who is a high-level executive and has dealt with product design decision-making for a long time and understands market trends, three BIPD researchers who have engaged in product bionic design research and education for a long time, and six ordinary users (four males and four females, aged 25–30 years) who are young white-collar workers with certain purchasing potential. Informed consent was obtained from all subjects involved in the study. This experiment has been ethically reviewed by the School of Art and Archaeology of Hangzhou City University.
(2) Stimulus: It can be difficult for subjects to clearly identify the evaluation indicators without some concrete examples, so design scenarios and bionic products were prepared for all subjects.

Two task scenarios that simulate the actual BIPD were provided. Several different bionic designs of similar products are provided as references. Three categories of bionic design products were selected, and under each category, there are different cases for the subjects to compare. All pictures are from the internet. The first category is bio-inspired water cup products, including cactus cups, rooster teapots, cat leg cups, Starbucks cat claw cups, whale cups, lotus leaf cups, and bamboo cups. Cars were selected as the second product category, and car design is one of the design fields in which designers most like to apply bionic elements. The bio-inspired cars include the Mercedes-Benz car with gull-wing doors, the Mercedes-Benz boxfish concept car, the banana car, the shark car, the frog car, and the stag beetle concept car by bionic design master Luigi Colani. The third type of bionic product is chairs, with bionic sources including flowers, whales, snails, etc.

Two task scenarios are described as follows.

- Scenario 1: Design a bionic electric car suitable for children aged 3 to 6 years old;
- Scenario 2: Design a bionic sweeping robot for young urban white-collar workers

#### 3.1.2. Experimental Procedure

Interviews began by explaining the concept of BIPD and the goals of the investigation. The two scenarios and bionic products were provided to assist their understanding. Thereafter, the designers were asked to state the factors they consider when designing and evaluating bionic products. Whereas the decision-maker was allowed to express the factors he or she considered in the process of designing, producing, and launching bionic products, the ordinary users were encouraged to discuss their considerations when choosing a bionic product. And they can easily explain their thoughts by evaluating the bionic designs of products from the same category and giving reasons why they like and dislike them.

All subjects were encouraged to think in multiple dimensions. The interview time for each subject was between 10 and 15 min. During the interview, the subjects’ spoken responses were recorded and written down for subsequent analysis.

#### 3.1.3. Experimental Results and Analysis

The method of Kansei engineering was applied to analyze the data [44]. After the experiment, 16 original spoken records were recorded. Then, the evaluation items were extracted from the original spoken reports:

1. The subjects’ original corpus was translated and the spoken expressions were summarized in neutral language to define clear evaluation criteria. For instance, the statement ‘I don’t like spider chairs because I hate insects’, was translated into the evaluation index ‘whether the bionic creature meets the user’s preferences’;
2. Evaluation indexes with the same semantic meaning were merged into one evaluation item. For example, ‘whether the product is unique’ and ‘is the product different’ are all evaluations of product personality. A total of 25 original evaluation items were identified after merging related items;
3. The frequency of subjects that identified each evaluation item was determined. A given item was recorded only once for a given subject, even if it was mentioned several times during the interview (Table 2).

Table 2 shows the BIPD evaluation items and their frequency, arranged from high to low.

### 3.2. BIPD Evaluation Model Construction

#### 3.2.1. Experimental Design and Procedure

In order to construct a hierarchical evaluation system using the evaluation indicators obtained in Section 3.1, it is necessary to merge indicators with the same meaning, eliminate indicators that are difficult to evaluate, and describe the indicators in more precise language. Moreover, the evaluation indicators need to be classified and layered. The focus group method was used to conduct this experiment.

Six product design experts with more than eight years of design and research experience were recruited to participate in a focus group discussion synthesizing the interview results. And the experiment was carried out through the following steps:

1. Eliminate some evaluation items that cannot be directly judged from product images. Items such as ‘product quality’, ‘durability’, and ‘production feasibility’, which cannot be directly judged, were eliminated.
2. Use precise and concise language to comprehensively describe the content of the evaluation items in general terms. For example, the two merged items in (1) can be rephrased to be described as ‘bionic compatibility’, which means that the product and the creature are matched in terms of shape, function, environment, etc.
3. Stratify the evaluation items, grouping those expressing the same level into one category. Finally, the BIPD evaluation indicators were divided into four layers: the overall goal layer, the sub-goal layer, the criterion layer, and the evaluation layer. Thus, a BIPD evaluation model was established.

#### 3.2.2. Experimental Results

**Figure 2:**
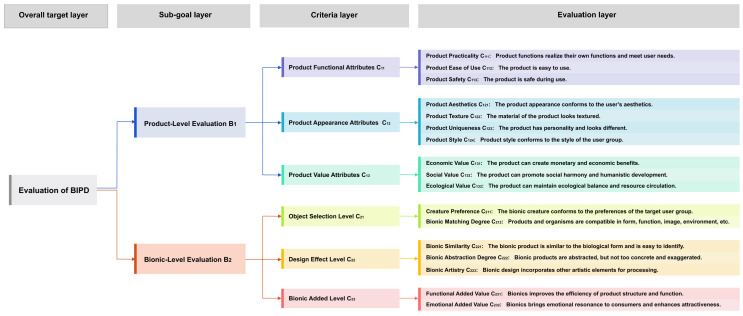
BIPD evaluation model.

After the experiment, a four-layer BIPD evaluation model was established. The four layers include the overall goal layer, sub-goal layer, criteria layer, and evaluation layer, which are explained as follows and illustrated in Figure 2.

1. Overall target layer A: evaluation of BIPD;
2. Sub-goal layer B: The overall goal layer is divided into sub-goal product-level evaluation $B_{1}$ and bionic-level evaluation $B_{2}$, each containing three evaluation criteria. $B_{1}$ mainly focuses on the general evaluation indicators of the product itself, while $B_{2}$ focuses on the requirements and concerns of bionic design;
3. Criteria layer C: Each sub-goal contains three criteria layers, which are the evaluation criteria under the goal. Product-level evaluation includes the functional attributes of the product $C_{11}$, appearance attributes of the product $C_{12}$, and value attributes of the product $C_{13}$. The bionic evaluation level includes the object selection level $C_{21}$ (organism and product selection), the bionic design effect level $C_{22}$, and the BIPD added value $C_{23}$.
4. Evaluation layer *D*: It is the evaluation index finally established by experts through discussion. The 17 indicators in the evaluation layer belong to different evaluation criteria, and they are explained in Figure 2.

### 3.3. BIPD Evaluation Model Weight Calculation

#### 3.3.1. Experimental Design and Procedure

According to the AHP method, in order to obtain a more objective and accurate calculation of the index weights among the different indicators of the BIPD evaluation model, the first steps were to conduct pairwise comparisons and create judgment matrices for each level. If we consider the upper-level criteria as K and the lower-level sub-criteria as $k_{1},\;k_{2},\cdots ,\;k_{n}$, it is possible to determine the weights indicating the relative importance of elements $k_{i}$ and $k_{j}$ to the criteria K. In AHP, the method for assessing the relative importance of indicators involves assigning numerical values and their reciprocals on a scale from 1 to 9 [28,55]. Table 3 shows the meaning of the scale for the judgment matrix used to evaluate the importance of the indicators (Saaty’s 1–9 scale).

The calculation formula for the judgment matrix is as follows:

(1) $$K={\left(k_{ij}\right)}_{n\times n}\;$$

**Figure 3:**
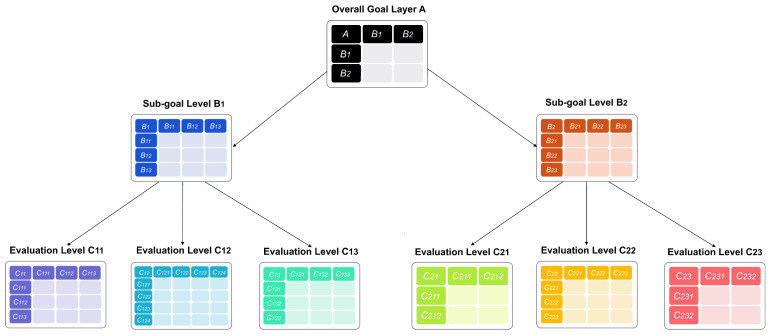
Pairwise comparison matrix tables.

The scale defined in Table 3 is used to construct judgment matrices of importance of each layer. Every two indicators in each layer are compared, so there are nine pairwise comparison matrix tables in total, as shown in Figure 3.

Six experts with different backgrounds were invited to conduct pairwise comparisons. They included two product design experts with more than six years of experience, two user research experts, and two bionic design research experts. The questionnaire included the purpose of the research, the BIPD evaluation hierarchical model (Figure 2), the evaluation index judgment matrix scale meaning table for BIPD (Table 3), and nine pairwise comparison matrices (Figure 3). The experts were asked to score the nine pairwise comparison matrices. The model weight will be calculated based on the score of each expert, and the final weight will be the average of the six experts. The comparison matrices for rating by each expert are shown in Appendix A.

#### 3.3.2. Weight Calculation Method

After obtaining the original pairwise comparison matrices of the six experts, the model weights were first calculated based on the data of each expert, and finally the weights of the six experts were averaged to obtain the final BIPD evaluation model weights.

There are several methods for calculating the model weight, including the geometric mean, arithmetic mean, eigenvector, and least-squares methods [34]. This article uses the eigenvector to calculate the weight, and there are three steps:

(1) Calculate the principal eigenvalue and its corresponding eigenvector

Assume $w={\left(w_{1},\cdots w_{n}\right)}^{T}$ is the weight vector obtained from the H-order comparison matrix and the pairwise comparison matrix A is the consistency matrix; then,

(2) $$A=\left[\begin{array}{cc} \begin{array}{cc} \frac{w_{1}}{w_{1}} & \frac{w_{1}}{w_{2}}\\ \frac{w_{2}}{w_{1}} & \frac{w_{2}}{w_{2}} \end{array} & \begin{array}{cc} \cdots & \frac{w_{1}}{w_{n}}\\ \cdots & \frac{w_{2}}{w_{n}} \end{array}\\ \begin{array}{cc} \vdots & \vdots \\ \frac{w_{n}}{w_{1}} & \frac{w_{n}}{w_{2}} \end{array} & \begin{array}{cc} & \vdots \\ \cdots & \frac{w_{n}}{w_{n}} \end{array} \end{array}\right]\;\;\;\;\;$$

If the rank of the matrix is 1, it satisfies

(3) AW=nW

n is the maximum eigenvalue of A, W is the eigenvector of A belonging to eigenvalue n, and all other eigenvalues of A are 0. The eigenvector corresponding to the maximum eigenvalue $\lambda_{max}$ of A is used, and the eigenvector W that satisfies the following formula is used as the approximate weight vector:

(4) $$AW=\lambda_{max}W\;\;\;$$

The goal is to obtain the largest eigenvalue $\lambda_{max}$ and its corresponding eigenvector [26]. In this study, the linear algebra software MATLAB R2019b was used to compute $\lambda_{max}$ and its corresponding eigenvector.

(2) Normalize the eigenvector

To ensure the weights sum to 1, the eigenvector is normalized. The eigenvector W corresponding to $\lambda_{max}$ represents the weights, with each component $w_{i}$ indicating the relative importance of criterion $k_{i}$. The normalized weight vector $W^{\prime }$ can be calculated as follows:

(5) $$W^{\prime }=\frac{w_{i}}{\sum_{i=1}^{n}w_{i}}\;\;\;$$

The normalized weight vector $W^{\prime }=\left(w_{1}^{\prime },w_{2}^{\prime },\cdots ,w_{n}^{\prime }\right)$ gives the relative weights of each criterion or alternative, indicating their importance in the decision-making process.

(3) Perform a single-level consistency test

In the AHP, pairwise comparisons are subjective, so they may contain inconsistencies. To ensure that the comparisons are reliable, consistency tests are conducted to make sure the calculated weights of each level are reasonable.

The first step is to calculate $\lambda_{max}$ using Equation (6).

(6) $$\lambda_{max}=\frac{1}{n}\sum_{i=1}^{n}\frac{{\left(AW\right)}_{i}}{w_{i}}$$

The second step is to calculate the Consistency Index (CI): The Consistency Index measures the degree of inconsistency in the matrix:

(7) $$CI=\frac{\lambda_{max}-n}{n-1}\;\;\;\;$$

where n is the order of the matrix, and a CI of 0 indicates perfect consistency; larger values indicate more inconsistency.

The third step is to calculate the Consistency Ratio (CR): The Consistency Ratio compares the CI to a Random Index (RI, as shown in Table 4), a value obtained from a table based on the size of the matrix [25]:

(8) $$CR=\frac{CI}{RI}$$

If CR<0.1, the level of inconsistency is acceptable. If CR≥0.1, the matrix has a high level of inconsistency, which is unacceptable, and the original expert needs to adjust the comparison matrix.

(4) Perform an overall consistency test

In multi-level AHP structures, local weights must be aggregated across levels to obtain the total weights of alternatives with respect to the overall goal. To ensure the logical consistency of the entire hierarchy, the overall consistency test evaluates the consistency of weight aggregation across all levels [25].

If level *X* contains m elements $Y_{1},$ $Y_{2},\;\cdots ,\;Y_{m}$ in the next layer Y, the corresponding local weights of these elements are $y_{1},\;y_{2},\;\cdots ,\;y_{m}$. Each element $Y_{i}$ has a single-level Consistency Index, $CI(Y_{i})$, and a corresponding Random Index $RI(Y_{i})$.

The overall Consistency Index CI(X) and the overall Random Index RI(X) for the hierarchy can then be calculated as weighted sums of the individual consistency indices for each element:

(9) $$CI\left(X\right)=\sum_{i=1}^{m}y_{i}\times CI\left(Y_{i}\right)\;\;\;\;\;\;\;$$

(10) $$RI\left(X\right)=\sum_{i=1}^{m}y_{i}\times CI\left(Y_{i}\right)\;\;\;\;\;\;\;$$

Similarly, the overall consistency is calculated using Formula (8).

#### 3.3.3. Experimental Results and Analysis

The normalized weight vector $W^{\prime }$, maximum eigenvalue $\lambda_{max}$, consistency index CI, and consistency ratio CR of each layer of each expert were calculated separately, as described in Section 3.3.2. For those cases where consistency was not passed, parts of the questionnaire that failed the consistency test were marked, and the experts were asked by phone to re-score the comparison matrix that failed the consistency test until it finally passed the consistency test.

The overall consistency ratio of the criterion layer to the sub-goal layer and the overall consistency ratio of the sub-goal layer to the total goal layer were calculated according to Formula (6). The consistency ratios of the six experts were all less than 0.1, and the total hierarchical ordering results met the consistency requirements.

**Figure 4:**
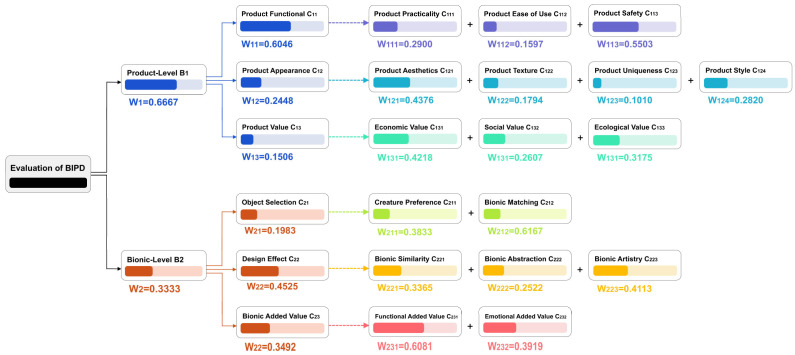
BIPD evaluation model’s comprehensive weight coefficient of each level.

The weight coefficients of all experts at each level are shown in Appendix B. The average weight coefficient of all experts is taken as the final comprehensive weight of each level [28]. The comprehensive weight coefficient of each level is shown in Figure 4 And the final weights of all evaluation items (indicator in the evaluation layer) in descending order are shown in Appendix C.

## 4. Model Validation

In this section, we will verify the effectiveness of the BIPD evaluation model through two evaluation cases: one is the evaluation of the bionic design of traditional products, and the other is the evaluation of the bionic design generated by AIGC.

### 4.1. Night Light Evaluation

According to the BIPD evaluation model and the comprehensive weight coefficients of each level, a BIPD evaluation method based on the AHP was constructed. When evaluating a bionic product design, stakeholders can score according to Appendix D, with a total of 17 evaluation indicators. The scores are 1 point (strongly disagree), 2 points (disagree), 3 points (average), 4 points (agree), and 5 points (strongly agree). The final score is then calculated based on the weight coefficients of each level of the BIPD evaluation model.

Assume that the subject scores the evaluation item $C_{kij}$ as $a_{kij}$; then, the subject’s comprehensive rating score M for the product can be calculated using Formula (11). And the weight coefficients are shown in Figure 4. The closer the score is to 5, the more satisfied the subject is with the bionic product.

(11) $$M=\sum_{q=1}^{k}\left(\sum_{i=1}^{n}\left(\sum_{j=1}^{m}a_{kij}\times W_{kij}\right)\times W_{ki}\right)\times W_{k}$$

**Figure 5:**
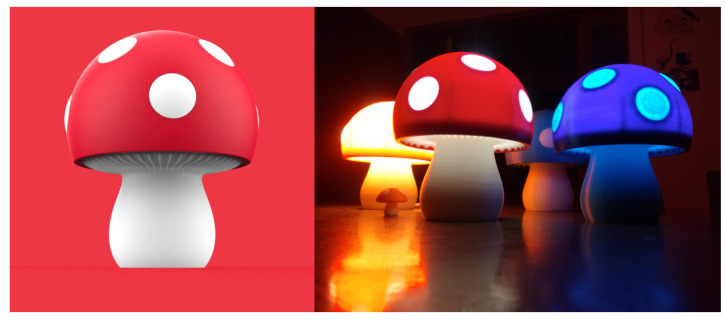
Mushroom night light by designer Zhang Fan.

**Figure 6:**
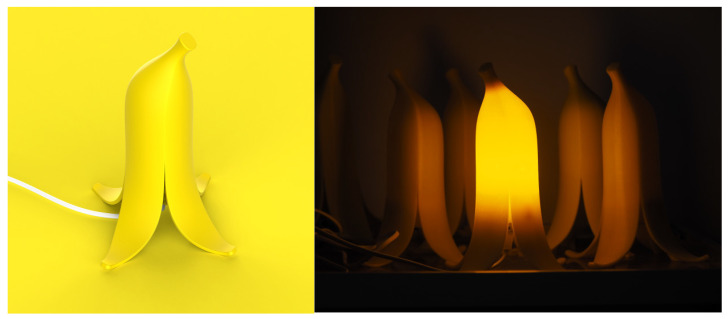
Banana night light by designer Zhang Fan.

Figure 5 and Figure 6 are two bionic night lights from a designer, and they were used as examples for BIPD evaluation. The following is an introduction to the two night lights.

(a) Mushroom night light: Inspired by mushrooms, the main body of this night light imitates the shape of a mushroom. The top umbrella-like cap is red, decorated with white dots, and the white part at the bottom resembles the stalk of a mushroom. The built-in light can present various colors such as warm yellow and blue.
(b) Banana night light: Designed with inspiration from bananas, the overall shape of this lamp imitates a peeled banana, with the main body being yellow. When lit, the light shines out from the inside. The structure that mimics the peeled part of the banana can support the lamp body, enabling it to stand stably.

Eight people who use night lights in their daily life or have the need to purchase night lights and six professional designers were invited to score the two night lights based on Appendix D. Informed consent was obtained from all subjects involved in the study. This experiment has been ethically reviewed by the School of Art and Archaeology of Hangzhou City University.

**Figure 7:**
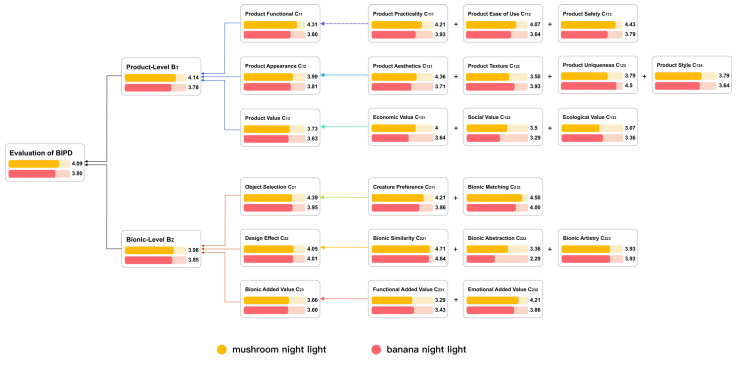
The scores for each layer of the two night lights. Note that the comprehensive score of the mushroom light night is higher than that of the banana night light.

The Cronbach’s alpha value of the questionnaire was 0.923, which indicated the reliability of the experimental data. The average scores of each item are considered the scores for the evaluation layer. After being substituted into Formula (9), the scores for the two night lights at the criteria layer, sub-goal layer, and overall goal layer were calculated, and the results are shown in Figure 7. The comprehensive scores for the mushroom night light and the banana night light are 4.09 and 3.80, respectively. The subjects were more satisfied with the bionic design of the mushroom night light.

According to Figure 7, the scores of 11 indicators (out of 18) of the mushroom night light are all higher than those of the banana night light. In particular, the scores of high-ranking indicators like product safety, product practicality, and product aesthetics are all relatively high. This may be because the shape of the banana night light is more special than that of ordinary night lights (it scores higher in product uniqueness), but the sense of security and ease of use conveyed by the product are lower.

Interestingly, before filling out the questionnaire, the subjects were asked which night light they preferred, and the number of people who chose the mushroom light and the number of people who chose the banana light were very close. The BIPD model explores hidden needs that are difficult for a single subject to think of and provides designers with judgments and improvement directions.

### 4.2. Camping Lights Evaluation

**Figure 8:**
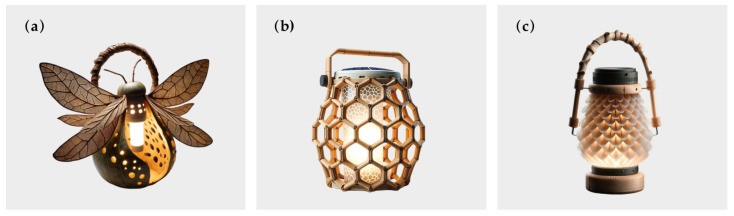
Camping Lights generated by Dall-E. (**a**) Firefly light. (**b**) Honeycomb light. (**c**) Pinecone light.

An industrial design student was recruited to use Dall-E to generate bionic camping lamps; three bionic camping lights were generated, as shown in Figure 8. The introductions of the three design cases are listed as follows.

(a) Firefly light: Inspired by fireflies, the main body mimics the abdomen of a firefly, crafted from natural gourds with a hollowed-out and perforated design. The semi-transparent wings feature vein-like structures. The natural–material handle at the top facilitates carrying and hanging, and the base ensures stability. The internal LED emulates the bioluminescence of fireflies.
(b) Honeycomb light: Deriving inspiration from honeycombs, the hexagonal frame replicates the honeycomb structure. It is made of lightweight and sustainable bamboo. The semi-transparent panels diffuse light. The solar panel on the top powers the internal LED, ensuring energy-efficiency and sustainability. The sturdy handle and flat base guarantee stability.
(c) Honeycomb light: Inspired by pine cones, the main body resembles a pine cone, with semi-transparent scales softening the light. The handle, resembling a tree branch, offers a natural and comfortable grip. The support structure and base are made of wood, ensuring stability. There is an operating mechanism above the base, and the internal LED provides illumination

**Figure 9:**
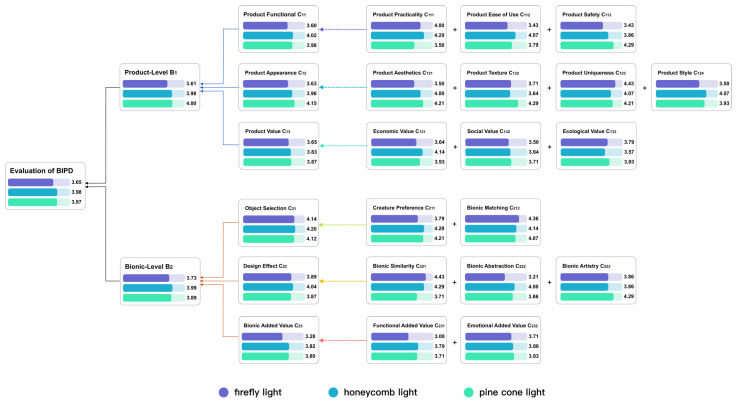
The scores for each layer of the three lights. Note that the comprehensive scores of the honeycomb light and the pinecone light are relatively high.

The student needed to choose from these three products for the next step of modeling and rendering, but each product had its own characteristics, making it difficult for her to decide. Therefore, eight camping enthusiasts and six professional designers were invited to score the designs based on Appendix D. The Cronbach’s alpha value of the questionnaire was 0.959, indicating the reliability of the experimental data, and the average values of each item were calculated. Finally, the values were substituted into Formula (9) for calculation. Assuming that the M values of the three bionic camping lights are M1 for the firefly light, M2 for the honeycomb light, and *M*3 for the pinecone light, then M1=3.65, M2=3.98, and M3=3.97. The scores for each layer of the three lights are shown in Figure 9. Therefore, the pinecone bionic light can be selected for the next step of the design. However, the honeycomb light also received a relatively high score, so it can be selected for modeling and design as well.

At the product level, the pinecone light scored slightly higher than the honeycomb light, and both were much higher than the firefly light. At the bionic level, the honeycomb light scored the highest, followed by the pinecone light, and the firefly light scored the lowest. The firefly light performs poorly in several indicators with higher weights, such as product safety, product aesthetics, functional added value, and ease of use. However, the firefly lamp has a higher score in product uniqueness and bionic matching. Improvements can be made in terms of product materials and aesthetics.

## 5. Discussion and Conclusions

This study has established a comprehensive evaluation system for Biologically Inspired Product Design (BIPD). The different considerations of various stakeholders when conducting BIPD evaluation were collected through an experiment at first. Then, evaluation indicators were extracted from original spoken reports. By applying the Analytic Hierarchy Process (AHP), a four-layer BIPD evaluation model was established, with accurate weights determined for each level.

The model sub-goal layer includes the product level and bionic level. From the weight coefficient, the product level is much more important than the bionic level. People first consider the safety, practicality, aesthetics, and functional added value of the product. Therefore, before designers carry out bionic creation, they should first meet the basic functional and appearance requirements of the product. As for the bionic evaluation indicators, bionic artistry is the indicator with the highest weight. People are more concerned about whether bionics can add artistry and beauty to the product—after all, nature is the source of beauty. Bionic similarity and bionic matching are two other indicators with higher weights at the bionic level, indicating that people are also concerned about whether bionic products are easy to identify and the relationship between the organism and the product itself.

The model can be effectively applied to the comparative evaluation of BIPD cases. It has the characteristics of fast scoring and accurate calculation, helping designers to screen and improve solutions in the conceptual design stage. In terms of sustainability, the BIPD evaluation model offers multiple benefits. Economically, it evaluates products at the pre-production stage, screens out unpromising solutions, saves costs, and ensures manufacturers’ long-term viability. Environmentally, it considers environmental factors in pre-production evaluation, with relevant indicators favoring eco-friendly products, reducing resource consumption and waste. Socially, by involving various stakeholders, it incorporates their views into the evaluation model, helps develop products meeting social needs, and assesses products’ impact on users and different social groups. Yet, future BIPD evaluations will need professionals to make judgments and create more detailed indicators for better product screening and improvement in these aspects.

However, the study also has some shortcomings. First of all, the experience and background of the invited subjects determined the evaluation items. A larger group of subjects could provide more objective and diverse indicators. Second, with the continuous development of the design industry, the evaluation indicators will evolve, giving the BIPD evaluation model a certain degree of timeliness. As new technologies, scenarios, and requirements emerge in the future, new evaluation indicators will also appear. These new indicators can be classified into the existing categories of the model, or new categories can be added. Different stakeholders would then be invited to score the importance of these new indicators, and the weights would be recalculated, enhancing the scalability of the model. Finally, the current model validation recruited target users and designers as test subjects to score. If relevant experts are invited to evaluate and score some indicators in the model, such as economic value and ecological value, more accurate feedback will be obtained. In the future, we will also be committed to exploring more quantifiable ways to score indicators.

## Figures and Tables

**Table 1:** Relevant Studies on BID evaluation.

Relevant Studies	Research Contents	Indicator Sources	Construction Methods	Indicators
Luo et al. [36]	Explore the inspiration of biological shapes on BID	Design experts	Discussion	Number of solutions, number of bionic elements, novelty, detail, abstraction
Wilson. et al. [37]	Explore the effects of biological examples in idea generation	Researchers	Previous literature	Novelty, variety
Vandevenne et al. [38]	Improve Ask Nature (a knowledge- based BID tool) to increase the innovation of BID	Researchers	Previous literature	Quantity, variety, novelty, quality
Luo et al. [44]	Predict users’ preferences for creatures through designers’ perceptual evaluation of creatures	Designers and ordinary users	Kansei engineering	It is mainly a perceptual evaluation of the organism and has nothing to do with the product.
Li, X. et al. [45]	Construct an inspiration library driven by user-perceived preference evaluation data for BID	BID designers and ordinary users	Kansei engineering	There is no specific mention in the article, mainly sentimental image vocabulary about organisms and products
Zhu et al. [46]	Evaluating biological inspiration for BID	Expert interview and literature analysis	A fuzzy rough number extended multi-criteria group decision-making (MCGDM)	Functional compatibility, constraint compatibility, attribute compatibility, behavioral compatibility, structural compatibility, novelty, manufacturing cost, power consumption, reliability
Aguilar-Planet et al. [47]	Multicriteria analysis of biomimetic methodologies.	Researchers	Not mentioned	Mainly an evaluation of bionic strategy tools, including the implementation time, gap between theory and practice, and so on.
Ours	Establish a scientific and sustainable BIPD evaluation system for industry	Product designers, sustainable experts, design company decision-makers, BID researchers, and ordinary users	Analytical hierarchy process (AHP)	Four-layer evaluation model with a total of 17 evaluation indicators; each indicator has a different weight.

**Table 2:** BIPD evaluation items.

$i_{t}$	Evaluation Items	Mentioned Frequency
$i_{1}$	Product practicality: whether the product functions meet the needs	14
$i_{2}$	Product aesthetics: whether the product meets aesthetic preferences	12
$i_{3}$	Product usability: is the product easy to operate and easy to use?	11
$i_{4}$	Biomimetic appropriateness: is it appropriate for biological elements to be used in this type of product?	11
$i_{5}$	Bionic similarity: whether the bionic product is similar to the biological form	11
$i_{6}$	Added value of bionics: in addition to form, whether product efficiency is improved through bionics	8
$i_{7}$	Product uniqueness: the product has personality and is unique	6
$i_{8}$	Product safety: the product is safe during use	5
$i_{9}$	Biological preference: the bionic creature conforms to the user’s preferences	5
$i_{10}$	Bionic appeal: do bionics bring emotional resonance to consumers?	5
$i_{11}$	Does the product style match the style of the user group?	4
$i_{12}$	Product added value: whether the product has other value besides its function	4
$i_{13}$	Bionic abstraction: whether bionic products are restrained and not too concrete and avant-garde	4
$i_{14}$	Product availability: is the product usable enough to complete its function?	3
$i_{15}$	Whether the bionic design has been artistically processed by adding other elements and textures	3
$i_{16}$	Bionic consistency: Are the feelings conveyed by the organism and the product consistent?	3
$i_{17}$	Emotional identification with the product	2
$i_{18}$	Realization of product economic value.	2
$i_{19}$	The feasibility of production, whether the structural materials are reasonable, and whether they can be produced	2
$i_{20}$	Product materials are sustainable and environmentally friendly.	2
$i_{21}$	Whether the bionic product is relevant and compatible with the environment in which it is used	2
$i_{22}$	Does the product have market value and is it suitable for mass production?	1
$i_{23}$	Product quality, durability	1
$i_{24}$	Product innovation	1
$i_{25}$	Products have social value	1

**Table 3:** Evaluation index judgment matrix scale meaning the table of BIPD (Saaty’s 1–9 scale).

$\mathrm{Relative}\;\mathrm{Importance}\;\mathrm{Value}\;(k_{i}/k_{j})$	Scale Meaning
1	Indicates that $k_{i}$ is as important as $k_{j}$.
3	Indicates that $k_{i}$ is more important than $k_{j}$.
5	Indicates that $k_{i}\;$ is much more important than $k_{j}$.
7	Indicates that $k_{i}\;$ is strongly more important than $k_{j}$.
9	Indicates that $k_{i}$ is absolutely more important than $k_{j}$.
2, 4, 6, 8	Indicates that when comparing $k_{i}$ and $k_{j}$, take the middle value of the above scale.
reciprocal	If $k_{ij}\;$ represent the relative importance of $k_{i}$ compared to $k_{j}$, then $k_{ji}$*=* 1/$k_{ij}$.

**Table 4:** Average Random Consistency Index from Tomas L. Saaty [51].

n	1	2	3	4	5	6	7	8	9	10
RI	0	0	0.52	0.89	1.11	1.25	1.35	1.40	1.45	1.49

## Data Availability

The original contributions presented in the study are included in the article, further inquiries can be directed to the corresponding authors.

## Appendix A

**Table A1:** Comparison matrices for rating by each expert.

Expert 1		
$A=\left[\begin{array}{cc} 1 & 1\\ 1 & 1 \end{array}\right]$		
$B_{1}=\left[\begin{array}{ccc} 1 & 3 & 9\\ 1/3 & 1 & 5\\ 1/9 & 1/5 & 1 \end{array}\right]$	$B_{2}=\left[\begin{array}{ccc} 1 & 1/2 & 5\\ 2 & 1 & 7\\ 1/5 & 1/7 & 1 \end{array}\right]$	
$C_{11}$ $=\left[\begin{array}{ccc} 1 & 2 & 2\\ 1/2 & 1 & 1/2\\ 1/2 & 2 & 1 \end{array}\right]$	$C_{12}=\left[\begin{array}{cc} \begin{array}{cc} 1 & 4\\ 1/4 & 1 \end{array} & \begin{array}{cc} 6 & 5\\ 5 & 2 \end{array}\\ \begin{array}{cc} 1/6 & 1/5\\ 1/5 & 1/2 \end{array} & \begin{array}{cc} 1 & 1/2\\ 2 & 1 \end{array} \end{array}\right]$	$C_{13}$ $=\left[\begin{array}{ccc} 1 & 4 & 5\\ 1/4 & 1 & 3\\ 1/5 & 1/3 & 1 \end{array}\right]$
$C_{21}=\left[\begin{array}{cc} 1 & 1/4\\ 4 & 1 \end{array}\right]$	$C_{22}=\left[\begin{array}{ccc} 1 & 4 & 3\\ 1/4 & 1 & 1\\ 1/3 & 1 & 1 \end{array}\right]$	$C_{23}=\left[\begin{array}{cc} 1 & 2\\ 1/2 & 1 \end{array}\right]$
Expert 2		
$A=\left[\begin{array}{cc} 1 & 3\\ 1/3 & 1 \end{array}\right]$		
$B_{1}=\left[\begin{array}{ccc} 1 & 3 & 3\\ 1/3 & 1 & 3\\ 1/3 & 1/3 & 1 \end{array}\right]$	$B_{2}=\left[\begin{array}{ccc} 1 & 1/3 & 1/7\\ 3 & 1 & 1/3\\ 7 & 3 & 1 \end{array}\right]$	
$C_{11}$ $=\left[\begin{array}{ccc} 1 & 3 & 1\\ 1/3 & 1 & 1/7\\ 1 & 7 & 1 \end{array}\right]$	$C_{12}=\left[\begin{array}{cc} \begin{array}{cc} 1 & 3\\ 1/3 & 1 \end{array} & \begin{array}{cc} 3 & 1\\ 1/3 & 1/2 \end{array}\\ \begin{array}{cc} 1/3 & 3\\ 1 & 2 \end{array} & \begin{array}{cc} 1 & 1/2\\ 2 & 1 \end{array} \end{array}\right]$	$C_{13}$ $=\left[\begin{array}{ccc} 1 & 1 & 1\\ 1 & 1 & 1\\ 1 & 1 & 1 \end{array}\right]$
$C_{21}=\left[\begin{array}{cc} 1 & 1/3\\ 3 & 1 \end{array}\right]$	$C_{22}=\left[\begin{array}{ccc} 1 & 3 & 3\\ 1/3 & 1 & 1/3\\ 1/3 & 3 & 1 \end{array}\right]$	$C_{23}=\left[\begin{array}{cc} 1 & 6\\ 1/6 & 1 \end{array}\right]$
Expert 3		
$A=\left[\begin{array}{cc} 1 & 5\\ 1/5 & 1 \end{array}\right]$		
$B_{1}=\left[\begin{array}{ccc} 1 & 7 & 9\\ 1/7 & 1 & 2\\ 1/9 & 1/5 & 1 \end{array}\right]$	$B_{2}=\left[\begin{array}{ccc} 1 & 1 & 9\\ 1 & 1 & 9\\ 1/9 & 1/9 & 1 \end{array}\right]$	
$C_{11}$ $=\left[\begin{array}{ccc} 1 & 1 & 1/9\\ 1 & 1 & 1/9\\ 9 & 9 & 1 \end{array}\right]$	$C_{12}=\left[\begin{array}{cc} \begin{array}{cc} 1 & 5\\ 1/5 & 1 \end{array} & \begin{array}{cc} 7 & 7\\ 3 & 3 \end{array}\\ \begin{array}{cc} 1/7 & 1/3\\ 1/7 & 1/3 \end{array} & \begin{array}{cc} 1 & 1/3\\ 3 & 1 \end{array} \end{array}\right]$	$C_{13}$ $=\left[\begin{array}{ccc} 1 & 3 & 7\\ 1/3 & 1 & 5\\ 1/7 & 1/5 & 1 \end{array}\right]$
$C_{21}=\left[\begin{array}{cc} 1 & 1/9\\ 9 & 1 \end{array}\right]$	$C_{22}=\left[\begin{array}{ccc} 1 & 1 & 3\\ 1 & 1 & 3\\ 1/3 & 1/3 & 1 \end{array}\right]$	$C_{23}=\left[\begin{array}{cc} 1 & 7\\ 1/7 & 1 \end{array}\right]$
Expert 4		
$A=\left[\begin{array}{cc} 1 & 3\\ 1/3 & 1 \end{array}\right]$		
$B_{1}=\left[\begin{array}{ccc} 1 & 2 & 5\\ 1/2 & 1 & 7\\ 5 & 7 & 1 \end{array}\right]$	$B_{2}=\left[\begin{array}{ccc} 1 & 1/3 & 1/7\\ 3 & 1 & 1/5\\ 7 & 5 & 1 \end{array}\right]$	
$C_{11}$ $=\left[\begin{array}{ccc} 1 & 1/3 & 1/5\\ 3 & 1 & 1/5\\ 5 & 3 & 1 \end{array}\right]$	$C_{12}=\left[\begin{array}{cc} \begin{array}{cc} 1 & 1/3\\ 3 & 1 \end{array} & \begin{array}{cc} 3 & 1/7\\ 3 & 1/5 \end{array}\\ \begin{array}{cc} 1/3 & 1/3\\ 7 & 5 \end{array} & \begin{array}{cc} 1 & 1/7\\ 7 & 1 \end{array} \end{array}\right]$	$C_{13}$ $=\left[\begin{array}{ccc} 1 & 1/2 & 1/5\\ 2 & 1 & 1/3\\ 5 & 3 & 1 \end{array}\right]$
$C_{21}=\left[\begin{array}{cc} 1 & 1/3\\ 3 & 1 \end{array}\right]$	$C_{22}=\left[\begin{array}{ccc} 1 & 1/3 & 1/5\\ 3 & 1 & 1/3\\ 5 & 3 & 1 \end{array}\right]$	$C_{23}=\left[\begin{array}{cc} 1 & 1/5\\ 5 & 1 \end{array}\right]$
Expert 5		
$A=\left[\begin{array}{cc} 1 & 1\\ 1 & 1 \end{array}\right]$		
$B_{1}=\left[\begin{array}{ccc} 1 & 1 & 1\\ 1 & 1 & 1\\ 1 & 1 & 1 \end{array}\right]$	$B_{2}=\left[\begin{array}{ccc} 1 & 1/5 & 1/3\\ 5 & 1 & 2\\ 3 & 1/2 & 1 \end{array}\right]$	
$C_{11}$ $=\left[\begin{array}{ccc} 1 & 3 & 1\\ 1/3 & 1 & 1/3\\ 1 & 3 & 1 \end{array}\right]$	$C_{12}=\left[\begin{array}{cc} \begin{array}{cc} 1 & 3\\ 1/3 & 1 \end{array} & \begin{array}{cc} 3 & 1\\ 1/3 & 1/3 \end{array}\\ \begin{array}{cc} 1/3 & 3\\ 1 & 3 \end{array} & \begin{array}{cc} 1 & 1/3\\ 3 & 1 \end{array} \end{array}\right]$	$C_{13}$ $=\left[\begin{array}{ccc} 1 & 1 & 3\\ 1 & 1 & 2\\ 1/3 & 1/2 & 1 \end{array}\right]$
$C_{21}=\left[\begin{array}{cc} 1 & 5\\ 1/5 & 1 \end{array}\right]$	$C_{22}=\left[\begin{array}{ccc} 1 & 1/3 & 1/5\\ 3 & 1 & 1/2\\ 5 & 2 & 1 \end{array}\right]$	$C_{23}=\left[\begin{array}{cc} 1 & 1/3\\ 3 & 1 \end{array}\right]$
Expert 6		
$A=\left[\begin{array}{cc} 1 & 9\\ 1/9 & 1 \end{array}\right]$		
$B_{1}=\left[\begin{array}{ccc} 1 & 1/3 & 1/5\\ 1/5 & 1 & 1/5\\ 1/5 & 7 & 1 \end{array}\right]$	$B_{2}=\left[\begin{array}{ccc} 1 & 1/5 & 1/3\\ 3 & 1 & 1/3\\ 1 & 5 & 1 \end{array}\right]$	
$C_{11}$ $=\left[\begin{array}{ccc} 1 & 5 & 1/7\\ 1/5 & 1 & 1/7\\ 7 & 7 & 1 \end{array}\right]$	$C_{12}=\left[\begin{array}{cc} \begin{array}{cc} 1 & 3\\ 1/3 & 1 \end{array} & \begin{array}{cc} 5 & 3\\ 5 & 3 \end{array}\\ \begin{array}{cc} 1/3 & 1/5\\ 1/3 & 1/5 \end{array} & \begin{array}{cc} 1 & 3\\ 1 & 1 \end{array} \end{array}\right]$	$C_{13}$ $=\left[\begin{array}{ccc} 1 & 5 & 1/5\\ 1/5 & 1 & 1/5\\ 1 & 9 & 1 \end{array}\right]$
$C_{21}=\left[\begin{array}{cc} 1 & 5\\ 1/5 & 1 \end{array}\right]$	$C_{22}=\left[\begin{array}{ccc} 1 & 1 & 1/5\\ 5 & 1 & 1/5\\ 5 & 5 & 1 \end{array}\right]$	$C_{23}=\left[\begin{array}{cc} 1 & 5\\ 1/5 & 1 \end{array}\right]$

## Appendix B

**Table A2:** Comparison matrix weight coefficient of overall target layer A.

Expert Number	$W_{1}$	$W_{2}$	$\lambda_{max}$	CI	CR
1	0.5000	0.5000	2	0.0000	0.0000
2	0.7500	0.2500	2	0.0000	0.0000
3	0.8333	0.1667	2	0.0000	0.0000
4	0.7500	0.2500	2	0.0000	0.0000
5	0.5000	0.5000	2	0.0000	0.0000
6	0.6667	0.3333	2	0.0000	0.0000

**Table A3:** Comparison matrix weight coefficient of product level evaluation $B_{1}$.

Expert Number	$W_{11}$	$W_{12}$	$W_{13}$	$\lambda_{max}$	CI	CR
1	0.6716	0.2654	0.0629	3.0291	0.0145	0.0279
2	0.6000	0.2000	0.2000	3.0000	0.0000	0.0000
3	0.7928	0.1312	0.0760	3.0217	0.0209	0.0209
4	0.5816	0.3090	0.1095	3.0037	0.0018	0.0036
5	0.3333	0.3333	0.3333	3.0000	0.0000	0.0000
6	0.6483	0.2297	0.1220	3.0037	0.0018	0.0036

**Table A4:** Comparison matrix weight coefficient of bionic level evaluation $B_{2}$.

Expert Number	$W_{21}$	$W_{22}$	$W_{23}$	$\lambda_{max}$	CI	CR
1	0.3332	0.5917	0.0751	3.0142	0.0071	0.0136
2	0.0879	0.2426	0.6694	3.0070	0.0035	0.0068
3	0.4737	0.4737	0.0526	3.0000	0.0000	0.0000
4	0.0810	0.1884	0.7306	3.0649	0.0324	0.0624
5	0.1095	0.5816	0.3090	3.0037	0.0018	0.0036
6	0.1047	0.6370	0.2583	3.0385	0.0193	0.0370

**Table A5:** Comparison matrix weight coefficient of product functional $C_{11}$.

Expert Number	$W_{111}$	$W_{112}$	$W_{113}$	$\lambda_{max}$	CI	CR
1	0.4934	0.1958	0.3108	3.0536	0.0268	0.0516
2	0.3879	0.0975	0.5146	3.0803	0.0401	0.0772
3	0.0909	0.0909	0.8182	3.0000	0.0000	0.0000
4	0.1095	0.3090	0.5816	3.0037	0.0018	0.0036
5	0.4286	0.1429	0.4286	3.0000	0.0000	0.0000
6	0.2297	0.1220	0.6483	3.0037	0.0018	0.0036

**Table A6:** Comparison matrix weight coefficient of product appearance $C_{12}$.

Expert Number	$W_{121}$	$W_{122}$	$W_{123}$	$W_{124}$	$\lambda_{max}$	CI	CR
1	0.5956	0.2246	0.0645	0.1153	4.1267	0.0422	0.0475
2	0.3847	0.1098	0.1933	0.3121	4.1752	0.0584	0.0656
3	0.6471	0.1908	0.0590	0.1032	4.2281	0.0760	0.0854
4	0.1165	0.2458	0.0535	0.5841	4.0850	0.0283	0.0318
5	0.3679	0.0956	0.1686	0.3679	4.1545	0.0515	0.0579
6	0.5140	0.2095	0.0670	0.2095	4.0973	0.0324	0.0364

**Table A7:** Comparison matrix weight coefficient of product value attribute $C_{13}$.

Expert Number	$W_{131}$	$W_{132}$	$W_{133}$	$\lambda_{max}$	CI	CR
1	0.6738	0.2255	0.1007	3.0858	0.0429	0.0825
2	0.3333	0.3333	0.3333	3.0000	0.0000	0.0000
3	0.6491	0.2790	0.0719	3.0649	0.0324	0.0624
4	0.1220	0.2297	0.6483	3.0037	0.0018	0.0036
5	0.4434	0.3874	0.1692	3.0183	0.0091	0.0176
6	0.3090	0.1095	0.5816	3.0037	0.0018	0.0036

**Table A8:** Comparison matrix weight coefficient of object selection level $C_{21}$.

Expert Number	$W_{211}$	$W_{212}$	$\lambda_{max}$	CI	CR
1	0.2000	0.8000	2	0.0000	0.0000
2	0.2500	0.7500	2	0.0000	0.0000
3	0.1000	0.9000	2	0.0000	0.0000
4	0.2500	0.7500	2	0.0000	0.0000
5	0.8333	0.1667	2	0.0000	0.0000
6	0.6667	0.3333	2	0.0000	0.0000

**Table A9:** Comparison matrix weight coefficient of design effect level $C_{22}$.

Expert Number	$W_{221}$	$W_{222}$	$W_{223}$	$\lambda_{max}$	CI	CR
1	0.6337	0.1744	0.1919	3.0092	0.0046	0.0088
2	0.6000	0.2000	0.2000	3.0000	0.0000	0.0000
3	0.4286	0.4286	0.1429	3.0000	0.0000	0.0000
4	0.1047	0.2583	0.6370	3.0385	0.0193	0.0370
5	0.1095	0.3090	0.5816	3.0037	0.0018	0.0036
6	0.1429	0.1429	0.7143	3.0000	0.0000	0.0000

**Table A10:** Comparison matrix weight coefficient of bionic added value $C_{23}$.

Expert Number	$W_{231}$	$W_{232}$	$\lambda_{max}$	CI	CR
1	0.6667	0.3333	2	0.0000	0.0000
2	0.8571	0.1429	2	0.0000	0.0000
3	0.8750	0.1250	2	0.0000	0.0000
4	0.1667	0.8333	2	0.0000	0.0000
5	0.2500	0.7500	2	0.0000	0.0000
6	0.8333	0.1667	2	0.0000	0.0000

## Appendix C

**Table A11:** Final weights of all evaluation items (Evaluation layer) in descending order.

Number	Evaluation Item	Weight (%)
$C_{113}$	Product Safety: The product is safe during use.	22.18
$C_{111}$	Product Practicality: Product functions realize their own functions and meet user needs.	11.69
$C_{121}$	Product Aesthetics: The product appearance conforms to the user’s aesthetics.	7.14
$C_{231}$	Functional Added Value: Bionics improve the efficiency of product structure and function.	7.08
$C_{112}$	Product Ease of Use: The product is easy to use.	6.44
$C_{223}$	Bionic Artistry: Bionic design incorporates other artistic elements for processing.	6.20
$C_{221}$	Bionic similarity: The bionic product is similar to the biological form and is easy to identify.	5.08
$C_{124}$	Product Style: The product style conforms to the style of the user group.	4.60
$C_{232}$	Emotional added value C: Bionics bring emotional resonance to consumers and enhances attractiveness.	4.56
$C_{131}$	Economic Value: The product can create monetary and economic benefits.	4.24
$C_{212}$	Bionic Matching: Products and organisms are compatible in form, function, image, environment, etc.	4.08
$C_{222}$	Bionic Abstraction Degree: Bionic products are abstracted but not too concrete and exaggerated.	3.80
$C_{133}$	Ecological Value: The product can maintain ecological balance and resource circulation.	3.19
$C_{122}$	Product Texture: The material of the product looks textured.	2.93
$C_{132}$	Social Value: The product can promote social harmony and humanistic development.	2.62
$C_{211}$	Creature Preference: The bionic creature conforms to the preferences of the target user group.	2.53
$C_{123}$	Product Uniqueness: The product has personality and looks different.	1.64

## Appendix D

**Table A12:** BIPD evaluation scale.

Number	Evaluation Item	Score				
$C_{111}$	Product Practicality: Product functions realize their own functions and meet user needs.	1	2	3	4	5
$C_{112}$	Product Ease of Use: The product is easy to use.	1	2	3	4	5
$C_{113}$	Product Safety: The product is safe during use.	1	2	3	4	5
$C_{121}$	Product Aesthetics: The product appearance conforms to the user’s aesthetics.	1	2	3	4	5
$C_{122}$	Product Texture: The material of the product looks textured.	1	2	3	4	5
$C_{123}$	Product Uniqueness: The product has personality and looks different.	1	2	3	4	5
$C_{124}$	Product Style: The product style conforms to the style of the user group.	1	2	3	4	5
$C_{131}$	Economic Value: The product can create monetary and economic benefits.	1	2	3	4	5
$C_{132}$	Social Value: The product can promote social harmony and humanistic development.	1	2	3	4	5
$C_{133}$	Ecological Value: The product can maintain ecological balance and resource circulation.	1	2	3	4	5
$C_{211}$	Creature Preference: The bionic creature conforms to the preferences of the target user group.	1	2	3	4	5
$C_{212}$	Biomimetic Bionic Matching: Products and organisms are compatible in form, function, image, environment, etc.	1	2	3	4	5
$C_{221}$	Bionic similarity: The bionic product is similar to the biological form and is easy to identify.	1	2	3	4	5
$C_{222}$	Bionic Abstraction Degree: Bionic products are abstracted, but not too concrete and exaggerated.	1	2	3	4	5
$C_{223}$	Bionic Artistry: Bionic design incorporates other artistic elements for processing.	1	2	3	4	5
$C_{231}$	Functional Added Value: Bionics improve the efficiency of the product structure and function.	1	2	3	4	5
$C_{232}$	Emotional added value C: Bionics bring emotional resonance to consumers and enhance attractiveness.	1	2	3	4	5
